# A Batteryless, Wireless Strain Sensor Using Resonant Frequency Modulation

**DOI:** 10.3390/s18113955

**Published:** 2018-11-15

**Authors:** Kyeong Jae Lee, Namsun Chou, Sohee Kim

**Affiliations:** 1 Department of Robotics Engineering, Daegu Gyeongbuk Institute of Science and Technology (DGIST), Daegu 42988, Korea; kj.lee@dgist.ac.kr; 2 Center for BioMicroSystems, Korea Institute of Science and Technology (KIST), Seoul 02792, Korea; nschou@kist.re.kr

**Keywords:** batteryless strain sensor, wireless strain sensor, resonant frequency modulation, Ecoflex

## Abstract

In this study, we demonstrated the feasibility of a wireless strain sensor using resonant frequency modulation through tensile impedance test and wireless sensing test. To achieve a high stretchability, the sensor was fabricated by embedding a copper wire with high conductivity in a silicone rubber with high stretchability, in which the resonant frequency can be modulated according to changes in strain. The characteristics of the sensor and the behavior of wireless sensing were calculated based on equations and simulated using finite element method. As the strain of the sensor increased, the inductance increased, resulting in the modulation of resonant frequency. In experimental measurement, as the strain of the sensor increased from 0% to 110%, its inductance was increased from 192 nH to 220 nH, changed by 14.5%, and the resonant frequency was shifted from 13.56 MHz to 12.72 MHz, decreased by 6.2%. It was demonstrated that using the proposed sensor, strains up to 110% could be detected wirelessly up to a few centimeters.

## 1. Introduction

Stomach cancer is one of the common cancers in Northeast Asian male population [1,2]. The general treatment is gastrectomy, which removes the stomach or the small intestine partially or totally [3,4,5]. After surgery, the stomach motility is observed to check normal gastric emptying functions using several clinical methods such as gastric emptying time measurement, ultrasound, scintigraphy, and gastroscopy [6,7,8]. Recently, methods such as electrogastrography (EGG) and magnetogastrography (MGG) have been introduced to quantitatively measure the stomach motility. However, in case of EGG, the electrodes must be attached onto the operated skin of the patient [9]. In case of MGG, it can be measured only in the place where the huge facility such as Superconducting Quantum Interference Device (SQUID) is located [10]. Both methods are not adequate to continuously measure the stomach condition right after the surgery.

**Figure 1:**
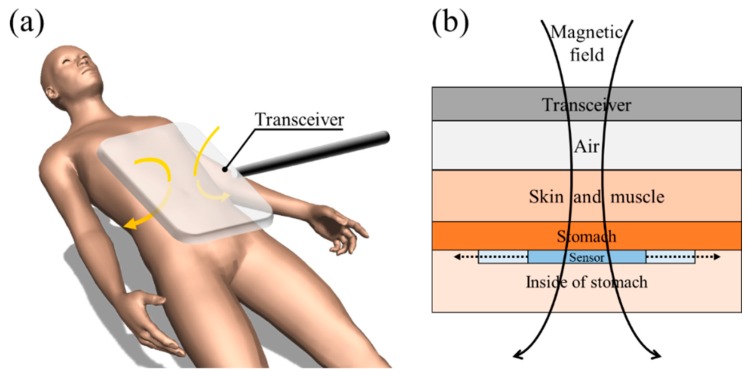
Schematic of the proposed sensor in a wireless sensing application. (**a**) Schematic illustration of the transceiver covering the entire abdomen to wirelessly measure the strain of the sensor attached to the stomach and (**b**) its cross-sectional view.

As a potential alternative to those methods, we propose a sensor that can be attached to the stomach directly and detect the stomach deformation wirelessly. Figure 1 illustrates the schematic of monitoring the stomach motility using the proposed sensor. The change in resonant frequency of the sensor could be measure wirelessly by a transceiver with a sufficiently large diameter to minimize the misalignment effect. The wireless strain sensor is attached to the stomach during surgery by stitching, for instance. When the stomach is active, the sensor deforms. The applied strain to the sensor causes the change in inductance, thereby shifting the resonant frequency of the sensor. The transceiver located outside the body senses the change in resonant frequency wirelessly by inductive coupling, and therefore the stomach deformation can be detected.

Several studies have reported sensors that demonstrated the feasibility of measuring the strain wirelessly. Kim et al. developed an LC tag-based RFID tag using silver nano-ink [11]. It operates in the 1.5 to 1.6 GHz frequency range, showing that as the tensile strain increases, the resonant frequency decreases within 4% of strain change. Song et al. developed a microstrip patch antenna made of silver nanowires and polydimethylsiloxane (PDMS), which operates at 2.9 to 3.1 GHz and the resonant frequency increases linearly with increasing strain within 15% of change [12]. Lazarus et al. proved that the modulation of resonant frequency caused by changes in strain can be measured wirelessly using a single-turn coil made of galistan and silicone rubber, which operates at frequencies of 0.97 to 1.29 MHz within 80% of strain change [13].

Unlike these previously developed sensors, the proposed sensor aims to eventually measure the stomach motility, which can stretch up to 100% and more [14]. To develop such a highly stretchable and wirelessly functioning strain sensor, the conducting patterns must have a low DC resistance enough to operate as a coil and at the same time, should have sufficient stretchability [15]. These two requirements are however contradictory, as outlined in many previous studies that the high stretchability can only be achieved by employing very thin conducting lines on very thin substrates [16,17,18]. Thus, we developed the wireless strain sensor that is patterned with a copper wire in a serpentine shape, based on a flexible substrate to achieve the higher stretchability that can cover the range of stomach motility as well as the wireless functionality.

## 2. Materials and Methods

### 2.1. Fabrication of the Sensor

**Figure 2:**
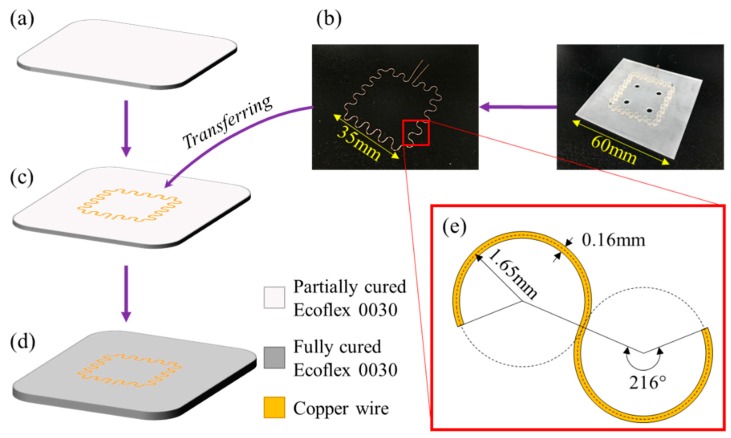
Fabrication process of the wireless strain sensor: (**a**) curing the substrate partially, (**b**) patterning the copper wire in serpentine shape, (**c**) transferring the pattern and (**d**) curing the device completely. (**e**) Detailed design of the serpentine pattern of the coil.

**Figure 3:**
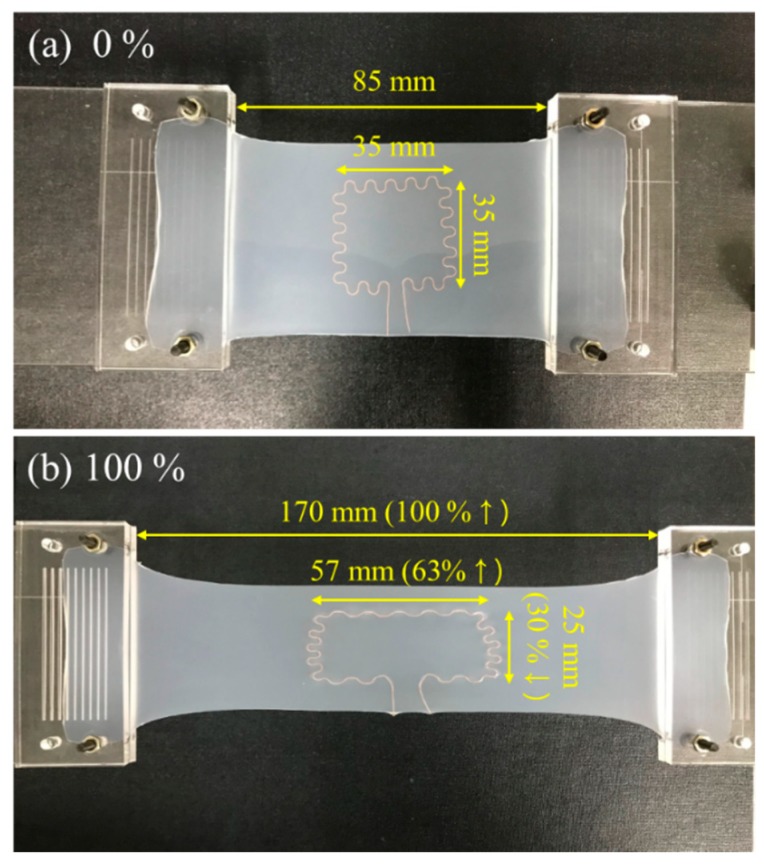
Photographs of the wireless strain sensor in (**a**) non-stretched and (**b**) 100% stretched states.

Figure 2 illustrates the fabrication process of the sensor. In the whole process, no specific semiconductor fabrication equipment was required. Ecoflex was chosen as the substrate material since it is flexible, highly stretchable and biocompatible [19,20,21]. Ecoflex mixture of part A and part B (Ecoflex 00-30, Smooth-On Inc., Easton, PA, USA) was prepared at a ratio of 1:1. The 15 g of mixture was poured into a petri dish to make a 1 mm thick substrate and left for 10 min at room temperature to remove air bubbles and make the surface even. The substrate was then partially cured in an oven at 60 °C for 3 min. Partially cured substrates enabled copper wires to be easily embedded in them (Figure 2a). Prior to the transfer step, the wire pattern was prepared using a custom-made winding mold. A copper wire with a diameter of 160 μm was wounded manually on the pillars printed by a 3D printer (Projet 3500, 3D systems, Rock Hill, SC, USA) to make a serpentine pattern (Figure 2b). The coil pattern was transferred to the top of the partially cured substrate and then 15 g of silicone rubber mixture was poured again onto the substrate with the serpentine pattern transferred (Figure 2c). It was then cured completely in the 60 °C oven for 10 min. The resulted coil had a single turn in a serpentine pattern embedded in silicone rubber in a thickness of 2 mm (Figure 2d). The outer dimension of the fabricated coil was 35 mm × 35 mm, the radius and angle of a single arc were 1.57 mm and 216°, respectively, taken from the previous study to maximize the stretchability [22] (Figure 2e). Figure 3 shows the fabricated sensor in non-stretched and stretched states. When the sensor was stretched by 100% in one direction, the coil pattern was stretched by 63% in the same direction and shrunk by 30% in the orthogonal direction. The resistance and the inductance of the sensor was 1.45 ± 0.04 Ω and 192 ± 3 nH, respectively. The Young’s modulus of the sensor was measured to be 40.5 ± 1.1 kPa.

To make the sensor resonate at a specific frequency, additional capacitors were connected in parallel to the sensor. Ceramic capacitors were used as they have a low parasitic inductance [23]. 13.56 MHz was selected as the resonating frequency of the sensor since it belongs to the industrial, scientific and medical (ISM) bands. The capacitance value to tune the sensor to resonate at 13. 56 MHz was 708 pF on average.

### 2.2. Calculation of Sensor Inductance

**Figure 4:**
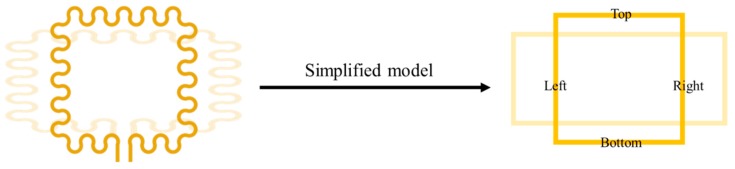
Simplification of the serpentine sensor coil as a rectangle to calculate the inductance.

The behavior of the proposed sensor was first predicted by simulations based on calculation. Due to the practical limitation that finite element method (FEM) simulation of electromagnetic fields is time consuming, we simplified the serpentine pattern as a straight line, so the shape change of the serpentine pattern could be simplified to the change in aspect ratio of a rectangle as illustrated in Figure 4. The inductance of the simplified model was calculated as the sum of self-inductances and mutual inductances of all line segments, based on the Greenhouse method [24]. In the Greenhouse method, the inductance is expressed as the sum of self-inductances and mutual inductances of each segments. The inductance *L* is given by

(1) $$\;L=\Sigma L_{s}+\Sigma M_{+}-\Sigma M_{-}\;$$

where *L_s_* is the self-inductance of each segment, and *M_+_* and *M_−_* are the positive and negative mutual inductance between segments, respectively. Since there is no segment combination in which current flows in the same direction, the sum of the positive mutual inductance is zero. The four segments used in the calculation are illustrated in Figure 4: left, right, top, and bottom segment.

Based on Equation (1), the inductance of the 1-turn rectangular coil has only two components of self-inductance *L_s_* and negative mutual inductance *M_−_*, and it can be calculated with the following equation:

(2) $$\;L=L_{s,\;Bottom}+L_{s,Left}+L_{s,Top}+L_{s,Right}+M_{-,\;Top,Bottom}+M_{-,Left,Right}\;$$

where $L_{s,\;Bottom}$, $L_{s,Left}$, $L_{s,\;Top}$, and $L_{s,Right}$ are the self-inductances of the bottom, left, top and right segment, respectively, and $M_{-,\;Top,Bottom}$ is the negative mutual inductance between the top and bottom segments, and $M_{-,Left,Right}$ is the negative mutual inductance between the left and right segments. *L_s_* and *M_−_* can be calculated by the following equations:

(3) $$\;L_{s}=0.002l\times \ln\left(\frac{2l}{l_{1}+l_{2}}+0.50049+\frac{l_{1}+l_{2}}{3l}\right)\;$$

(4) $$\;M_{-}=2l\left[\ln\left\{\frac{l}{d}+{\left(1+\frac{l^{2}}{d}\right)}^{\frac{1}{2}}\right\}-{\left(1+\frac{d^{2}}{l^{2}}\right)}^{\frac{1}{2}}+\left(\frac{d}{l}\right)\right]\;$$

where *l* is the length of the selected segment, *l*_1_ is the horizontal length of the coil, *l*_2_ is the vertical length of the coil, and *d* is the distance between the segment centers. The relationship between the length of the coil segments and the strain applied to the sensor is based on the measurement that when the sensor was stretched 100%, the length of the top and bottom segments were increased by 63% and the length of the left and right segments were decreased by 30% as illustrated in Figure 3. Based on this, *l*_1_ and *l*_2_ of the coil when a strain *ε* is applied to the whole sensor are expressed as below:

(5) $$\;l_{1}=1.63\varepsilon l_{1,0\%}\;$$

(6) $$\;l_{2}=0.7\varepsilon l_{2,0\%}\;$$

where *l*_1,0%_ is the horizontal length at 0% strain and *l*_2,0%_ is the vertical length at 0% strain. Thus, the inductance of the sensor when a strain is applied can be calculated using Equation (2), substituted by Equations (3) to (6). This calculation was implemented in MATLAB (MATLAB 2016b, Mathworks Inc., Natick, MA, USA). Based on this method, the relationship between the inductance and strain could be obtained by linear fitting based on the calculated inductance values, expressed by

(7) $$\;L\approx L_{0}+\alpha_{0}\varepsilon \;$$

where *L* is the inductance of the sensor, $\alpha_{0}$ is the stretching coefficient, *L*_0_ is the non-stretched inductance, and *ε* is the applied strain. As the sensor consisted of an LC circuit to resonate at a specific frequency, the resonant frequency *f* of which is calculated as:

(8) $$\;f\approx \frac{1}{2\pi \sqrt{\left(L_{0}+\alpha_{0}\varepsilon \right)C}}\;$$

where *C* is the additional tuning capacitance.

### 2.3. Finite Element Simulation of Wireless Sensing

**Figure 5:**
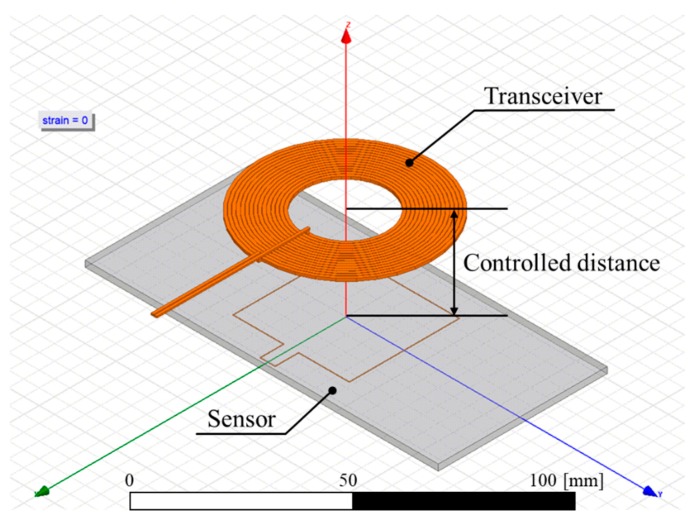
Simulation model of the sensor and the transceiver for wireless sensing. The distance between the sensor and the transceiver was changed from 10 mm to 50 mm, and the strain applied to the sensor was changed from 0% to 100%. In this figure, the transceiver is 20 mm away from the non-stretched sensor.

Before measuring the maximum distance that the sensor could operate wirelessly, finite element simulation using an electromagnetic simulator ANSYS HFSS (ANSYS Inc., Canonsburg, PA, USA) was performed to estimate the wireless working distance. Although the inductance of the sensor can be relatively easily calculated based on equations, it is not easy to mathematically derive the coupling coefficient between the sensor and the transceiver and thus, we simulated the wireless sensing using FEM. The simulation model is illustrated in Figure 5. The simplified geometry of the sensor was used, same as the model used in inductance calculation. Both the transceiver and the sensor were set to resonate at 13.56 MHz. The sensitivity of frequency shifting was defined as the gauge factor as follows:

(9) $$\;\mathrm{Gauge}\;\mathrm{Factor}\;=\frac{\Delta f/f_{0}}{\varepsilon }\;$$

where *f*_0_ is the resonant frequency at 0% strain, Δ*f* is the difference between *f*_0_ and the measured resonant frequency, and *ε* is the applied strain.

### 2.4. Experimental Setup

**Figure 6:**
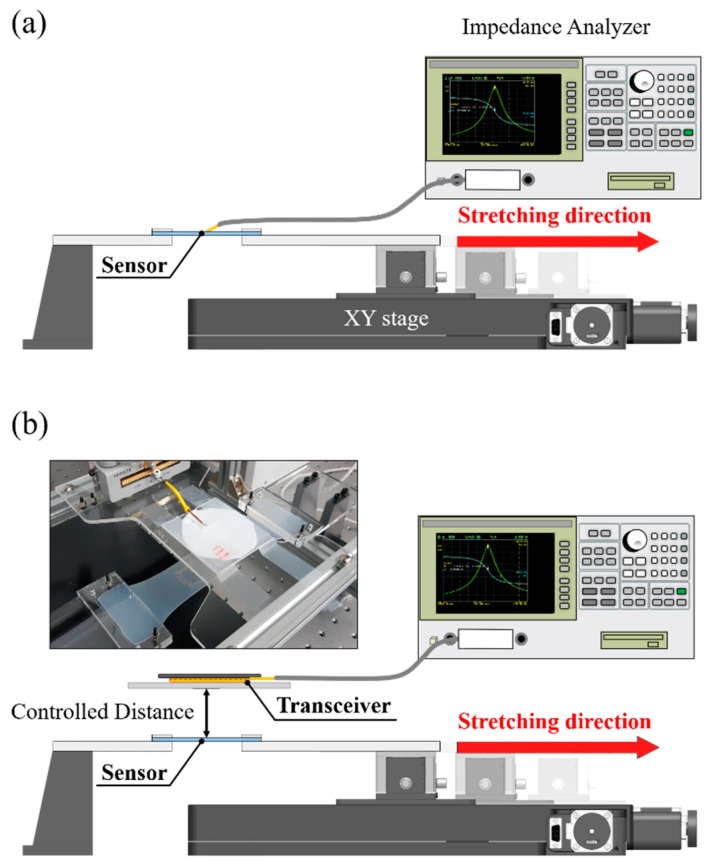
Experimental setup configurations for (**a**) tensile impedance testing and (**b**) wireless sensing.

To verify whether the change in sensor strain results in resonant frequency shifting, the experimental test bench was constructed as shown in Figure 6a. An impedance analyzer (4294A, Agilent, Santa Clara, CA, USA) was directly connected to the sensor to measure the impedance, and the strain applied to the sensor was controlled by an XY stage (CROSS 130-HSM, OWIS^®^, Staufen, Germany). Since the applied strain and the impedance should be measured at the same time, a LabVIEW-based program (LabVIEW 2016, National Instrument, Austin, TX, USA) was used to control the XY stage and the impedance analyzer simultaneously. Because the available travel range of the XY stage was limited, the sensor was stretched up to 110% at maximum. From each impedance measurement, the resistance, inductance, and capacitance of the sensor were evaluated using the equivalent circuit model, in which the resistance and inductance are connected in series and capacitance connected to those in parallel [25]. Figure 6b shows the experimental setup to observe whether the change in strain of the sensor can be measured wirelessly and to investigate the maximum distance of wireless detection. The deformation of the sensor was measured wirelessly using a transceiver, which was a circular planar coil with 15 turns, an outer diameter of 65 mm and a wire diameter of 1 mm, resulting in a self-inductance of 10 μH and the quality factor of 71. The impedance of the transceiver was measured as the strain applied to the sensor varied from 0% to 100%. The distance between the sensor and the transceiver was varied from 10 mm to 50 mm to observe the maximum distance for wireless sensing. The centers of the sensor and the transceiver were aligned manually at every measurement step. Since the purpose of the wireless sensing setup was to observe the modulation of resonant frequency at the transceiver with varying the vertical distance, the influence of coil misalignment was excluded.

## 3. Results

### 3.1. Tensile Impedance Test

**Figure 7:**
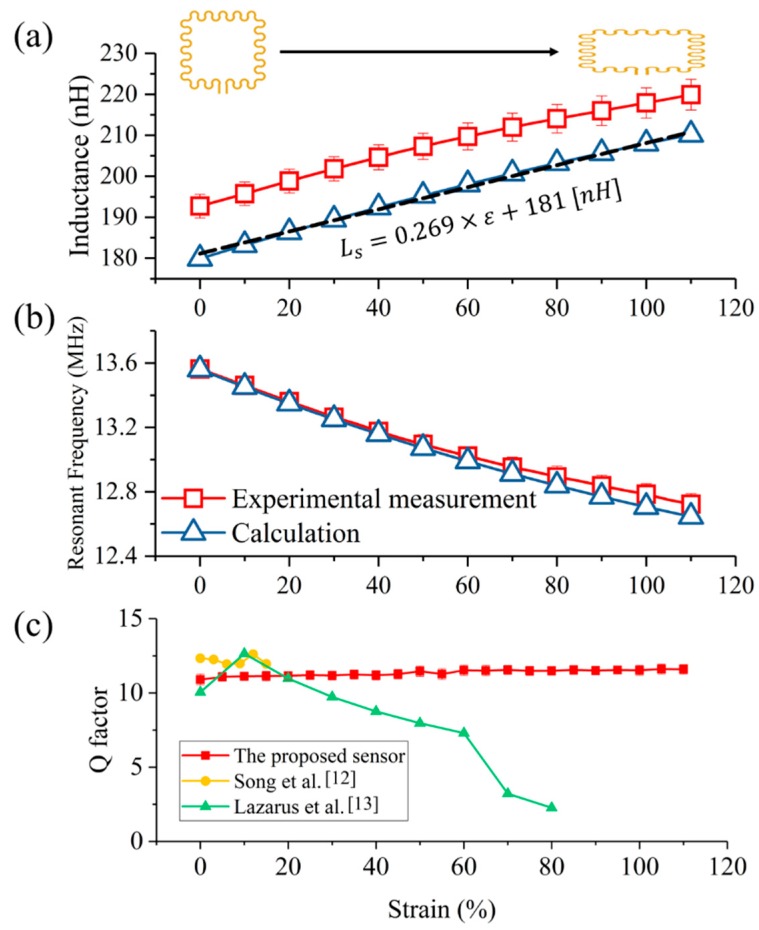
(**a**) Inductance change and (**b**) shift in resonant frequency of the sensor according to changes in strain, calculated using the Greenhouse method and measured experimentally. (**c**) Quality factor of the sensor as a function of strain, and compared with other sensors reported in previous studies [12,13].

The behavior of the proposed sensor was predicted by simulations based on calculation, and compared to measurements. As shown in Figure 7a, the calculated and measured results show that the inductance increased as the strain increased. The calculated inductance increased by 16.6% at 110% strain, and the measured inductance increased by 14.5%. The measured values were slightly higher than the calculated values, which was speculated to be caused by the parasitic inductances due to the serpentine structure and the additional measuring leads connected to the impedance analyzer. In Figure 7b, the resonant frequencies according to different strains are presented by using the calculated inductance, and compared with measurements. The calculated resonant frequency decreased by 6.7% with the change in strain of 110%, and the measured resonant frequency decreased by 6.2%. Both the measured and calculated resonant frequency decreased at a similar rate, as the inductance increased at a similar rate in both the measured and calculated cases. Figure 7c shows the quality factor (Q) of the sensor as a function of strain. The quality factor of the sensor was measured to be 10.9 ± 0.3 at 0% strain to 11.6 ± 0.3 at 110% strain, increased by 6.3%. For comparison with other studies, we calculated the Q of the wireless strain sensors reported in [12,13] based on the provided values such as resonant frequency, bandwidth, resistance, and inductance as those studies did not provide the Q explicitly. The fabricated sensor exhibited the Q similar to those of any stretchable strain sensors reported in the literature, up to 15% strain [12,13], but maintained a much higher Q for strains greater than 15%. This is thanks to the fabrication method of embedding the copper wire in thin silicone rubber sheet.

### 3.2. Wireless Sensing Test

**Figure 8:**
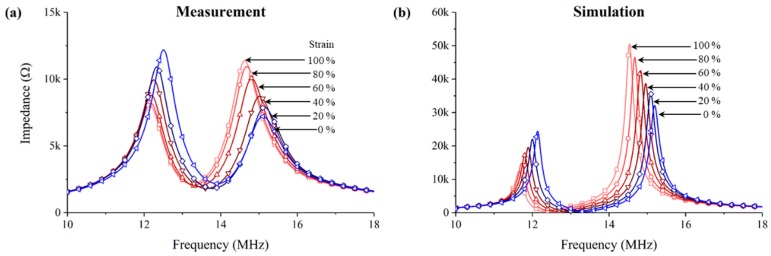
Experimentally measured and simulated impedance magnitudes of the transceiver at 10 mm away from the sensor. The frequency of the right peak was shifted (**a**) from 15.01 MHz at 0% strain to 14.59 MHz at 100% strain in experiment, and (**b**) from 15.31 MHz at 0% strain to 14.47 MHz at 100% strain in simulation.

Next, we tested the wireless strain sensing, and compared with simulations using electromagnetic simulator. Figure 8 compares the wireless sensing results from the measurement and simulation when the transceiver was 10 mm away from the sensor. Among the two peaks, the right peak was chosen as the resonant frequency of the transceiver, because the changes in frequency of the right peak showed a higher sensitivity than that of the left peak. The measured and simulated results show that the resonant frequency decreased as the sensor strain increased. Overall, the simulated impedance magnitudes were higher than the measured values. It appears to be due to the relatively high DC resistance of the sensor in measurements, caused by the serpentine shape, which however was not included in the simplified simulation model. Nevertheless, the degree of changes in the peak frequency of the simulation was similar to that of the measured results, so that the simulation results could be used to predict the maximum wireless working distance of the sensor.

**Figure 9:**
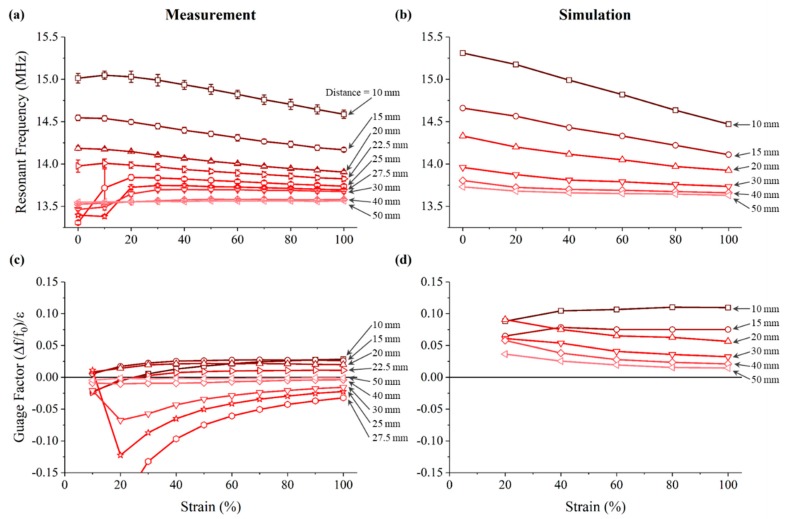
Experimentally measured and simulated results of wireless strain sensing. (**a**) Measured and (**b**) simulated resonant frequency modulation as the strain applied to the sensor increased, and the gauge factor of the transceiver calculated based on (**c**) the measured and (**d**) simulated resonant frequency.

The shifted resonant frequencies and the gauge factors of strain sensing according to different strains and different distances are summarized in Figure 9. Figure 9a shows the measured resonant frequency modulation detected at the transceiver wirelessly, and Figure 9c shows the calculated gauge factors. As the strain of the sensor increased, the resonant frequency of the transceiver decreased gradually. This decreasing trend could be seen only up to 22.5 mm distance. When measured at farther distances, the frequency converged to the resonant frequency of the transceiver itself, indicating that no measurable changes were detected. At 100% strain, the gauge factor decreased from 0.028 to 0.011 at 10 mm to 22.5 mm distance, converging to 0 at farther distances. Figure 9b shows the simulated resonant frequency at the transceiver, and the calculated gauge factors are shown in Figure 9d. The simulated results also showed that as the distance increased, the frequency converged to the self-resonant frequency of the transceiver. The detectable distance was observed to be between 20 mm and 30 mm, in good agreement with the experimental results. However, unlike the measured results, the simulated results did not show that the resonant frequency first increased at strains up to 20% at distances greater than 20 mm. This appears to be because the simplified simulation model did not include the additional DC resistance caused by the serpentine pattern and therefore did not fully reflect the decrease of inductive coupling. Nonetheless, the simulation results showed that the used FEM model could predict the wirelessly measurable distance reasonably well.

## 4. Discussion

To measure the motility of internal organs such as the stomach, a stretchable, batteryless and wireless strain sensor using resonant frequency modulation was investigated. This sensor can stretch up to 110% from its original length, shifting the resonant frequency by 6.2% at maximum around 13.56 MHz, which exceeds the strain ranges that have been measured wirelessly in previous studies [11,12,13]. Previous studies have shown that up to 80% strain can be measured wirelessly. However, to measure the stomach motility, the sensor must be able to detect a stretch of up to more than 100%, and thus, we have developed a sensor that can wirelessly measure strains up to 110%. The conductivity of the copper wire used in the present study (5.96 × 10^5^ S/cm) [26] is higher than the conductivity of the materials used in previous studies, by about 9 to 4000 times (silver nano-ink: 1.5 × 10^2^ S/cm [11], silver nanowire: 8.13 × 10^3^ S/cm [12], and eGaIn: 3.4 × 10^4^ S/cm [27]). In addition, since the Q increases as the coil resistance decreases [15], the sensor made of a copper wire developed in the present study has the advantage of maintaining a high Q for a wider range of strains than those reported in previous studies. This high Q of the proposed sensor was obtained through embedding the copper wire in silicone rubber sheet, unlike the other commonly employed methods to fabricate stretchable strain sensors based on thin metal films on thin substrates.

The sensor could detect the strain wirelessly at up to 22.5 mm distance by the used transceiver, although there is room for further improvement to increase the operating distance. Thus, our proposed sensor demonstrated that it satisfied the two contradictory requirements for wireless sensing of strains, namely, the high conductivity of the coil sensor and the high stretchability. Moreover, the maximum strain of the developed sensor was not inherently limited, but instead, originated from the limitation of the used experimental setup. One additional point to note is that we only investigated the resonant frequency modulation in response to strains in only one direction. For actual applications, however, bi-axial strains and bending would need to be applied. Previous studies show that there is a correlation between the diameter of the coil pattern and the wireless sensing performance due to bending. The wireless sensing performance is less affected by the degree of bending of the sensor when the diameter of the coil sensor is smaller [13,28,29]. Thus, to minimize the bending effect caused by the curved surface of the stomach, the outer length of the coil pattern would need to be minimized. Also, to increase the distance of wireless sensing and the sensitivity, parameter optimizations would be needed, which include the reduction of the sensor thickness, the increase in the number of turns of the sensor to increase the degree of inductive coupling between the sensor and the transceiver, and the increase of the outer diameter of the transceiver.

To apply the developed sensor in a real application, misalignment between the sensor and the transceiver should be overcome. To minimize the misalignment effect, the transceiver is suggested to be large enough to cover the entire abdomen as illustrated in Figure 1a. By creating a magnetic field as uniformly as possible throughout the abdomen, the sensor can keep relatively constant coupling with the external transceiver even if the exact location of the sensor is unknown. As shown in Figure 1b, the distance between the sensor and the transceiver can be determined by knowing the distance from the abdominal surface to the sensor, which could be measured by a physician during the surgery or by a medical imaging method. Therefore, it would be possible to detect the deformation of internal organs such as the stomach. To minimize the variations in stomach conditions and posture, the patient would be asked to stay with a constant posture, e.g., laying down on a bed, during the detection. Also, checking the stomach motility would be performed a few days after the gastrectomy, in which duration the food intake is generally restricted and thus, the stomach condition is expected to be quite constant. However, all such aspects need further investigation including animal experiments.

## References

[1] Jung K.-W., Won Y.-J., Kong H.-J., Oh C.-M., Cho H., Lee D.H., Lee K.H. (2015). Cancer Statistics in Korea: Incidence, Mortality, Survival, and Prevalence in 2012. Cancer Res. Treat..

[2] Chen W., Zheng R., Baade P.D., Zhang S., Zeng H., Bray F., Jemal A., Yu X.Q., He J. (2016). Cancer Statistics in China. CA Cancer J. Clin..

[3] Mochiki E., Kamiyama Y., Aihara R., Nakabayashi T., Asao T., Kuwano H. (2005). Laparoscopic assisted distal gastrectomy for early gastric cancer: Five years’ experience. Surgery.

[4] Choi S.H., Noh S.H., Min J.S., Lee K.S., Kim C.K. (1991). Clinical Analysis According to Reconstructive Type after Total Gastrectomy for Gastric Cancer. Ann. Surg. Treat. Res..

[5] Noh S.H., Yoo C.H., Kim Y., Kim C.B., Min J.S., Lee K.S. (1998). Results after a Gastrectomy of 2,603 Patients with Gastric Cancer: Analysis of Survival Rate and Prognostic Factor. Ann. Surg. Treat. Res..

[6] Jung H.J., Kim D.H., Kim D.H. (2008). Proximal Gastrectomy with Double Tract Reconstruction Using the Remnant Antrum in Early Upper Gastric Cancer. Ann. Surg. Treat. Res..

[7] Jeong I.-U., Song Y.-J., Yun H.-Y. (2000). Gastric-Emptying Patterns after Gastroduodenal Reconstruction. Ann. Surg. Treat. Res..

[8] Kim W., Jeon H.M., Hur H., Lee J.H., Won J.M. (2004). Jejunal Pouch Interposition (JPI) after Distal Gastrectomy. J. Korean Gastric Cancer Assoc..

[9] Komorowski D., Pietraszek S., Tkacz E., Provaznik I. (2015). The extraction of the new components from electrogastrogram (EGG), using both adaptive filtering and electrocardiographic (ECG) derived respiration signal. Biomed. Eng. Online.

[10] Estombelo-Montesco C.A., de Araujo D.B., Roque A.C., Moraes E.R., Barros A.K., Wakai R.T., Baffa O., Davies M.E., James C.J., Abdallah S.A. (2007). Extraction of gastric electrical response activity from magnetogastrographic recordings by DCA. Indep. Compon. Anal. Signal Sep. Proc..

[11] Kim J., Wang Z., Kim W.S. (2014). Stretchable RFID for wireless strain sensing with silver nano ink. IEEE Sens. J..

[12] Song L., Myers A.C., Adams J.J., Zhu Y. (2014). Stretchable and reversibly deformable radio frequency antennas based on silver nanowires. ACS Appl. Mater. Interfaces.

[13] Lazarus N., Meyer C.D., Turner W.J. (2015). A microfluidic wireless power system. RSC Adv..

[14] Egorov V.I., Schastlivtsev I.V., Prut E.V., Baranov A.O., Turusov R.A. (2002). Mechanical properties of the human gastrointestinal tract. J. Biomech..

[15] Neagu C.R., Jansen H.V., Smith A., Gardeniers J.G.E., Elwenspoek M.C. (1997). Characterization of a planar microcoil for implantable microsystems. Sens. Actuators A Phys..

[16] Rogers J.A., Someya T., Huang Y. (2010). Materials and mechanics for stretchable electronics. Science.

[17] Fan J.A., Yeo W.H., Su Y., Hattori Y., Lee W., Jung S.Y., Zhang Y., Liu Z., Cheng H., Falgout L. (2014). Fractal design concepts for stretchable electronics. Nat. Commun..

[18] Huang X., Liu Y., Cheng H., Shin W.J., Fan J.A., Liu Z., Lu C.J., Kong G.W., Chen K., Patnaik D. (2014). Materials and designs for wireless epidermal sensors of hydration and strain. Adv. Funct. Mater..

[19] (2013). Ecoflex Series.

[20] Salvatore G.A., Sülzle J., Dalla Valle F., Cantarella G., Robotti F., Jokic P., Knobelspies S., Daus A., Büthe L., Petti L. (2017). Biodegradable and Highly Deformable Temperature Sensors for the Internet of Things. Adv. Funct. Mater..

[21] Park G., Chung H.J., Kim K., Lim S.A., Kim J., Kim Y.S., Liu Y., Yeo W.H., Kim R.H., Kim S.S. (2014). Immunologic and tissue biocompatibility of flexible/stretchable electronics and optoelectronics. Adv. Healthc. Mater..

[22] Chou N., Lee J., Kim S. (2014). Large-sized out-of-plane stretchable electrodes based on poly-dimethylsiloxane substrate. Appl. Phys. Lett..

[23] Horowitz P., Winfield H. (2015). The Art of Electronics.

[24] Greenhouse H.M. (1974). Design of Planar Rectangular Microelectronic Inductors. IEEE Trans. Parts Hybrids Packag..

[25] Agilent Technologies (2003). Agilent 4294A Precision Impedance Analyzer Operation Manual.

[26] So J.H., Thelen J., Qusba A., Hayes G.J., Lazzi G., Dickey M.D. (2009). Reversibly deformable and mechanically tunable fluidic antennas. Adv. Funct. Mater..

[27] Hayes G.J., So J.-H., Qusba A., Dickey M.D., Lazzi G. (2012). Flexible Liquid Metal Alloy (EGaIn) Microstrip Patch Antenna. IEEE Trans. Antennas Propag..

[28] Shin G., Gomez A.M., Al-Hasani R., Jeong Y.R., Kim J., Xie Z., Banks A., Lee S.M., Han S.Y., Yoo C.J. (2017). Flexible Near-Field Wireless Optoelectronics as Subdermal Implants for Broad Applications in Optogenetics. Neuron.

[29] Kim J., Banks A., Xie Z., Heo S.Y., Gutruf P., Lee J.W., Xu S., Jang K.I., Liu F., Brown G. (2015). Miniaturized Flexible Electronic Systems with Wireless Power and Near-Field Communication Capabilities. Adv. Funct. Mater..

